# Complement-producing maternal microchimeric cells override infection susceptibility in complement-deficient murine offspring

**DOI:** 10.1172/JCI187001

**Published:** 2024-11-14

**Authors:** Giang Pham, Raymond E. Diep, Lucien H. Turner, David B. Haslam, Sing Sing Way

**Affiliations:** Division of Infectious Diseases, Center for Inflammation and Tolerance, Cincinnati Children’s Hospital Department of Pediatrics, University of Cincinnati College of Medicine, Cincinnati, Ohio, USA.

**Keywords:** Infectious disease, Reproductive biology, Bacterial infections, Genetic diseases, Organ transplantation

**To the Editor:** Long-term persistence of vertically transferred maternal cells occurs ubiquitously in mammalian offspring. The presence of these exceptionally rare maternal microchimeric cells (MMcs), with ensuing immunological tolerance to noninherited maternal antigen (NIMA), is associated with a variety of remarkable phenotypes including serological resistance to noninherited maternal HLA sensitization (1), improved long-term survival of NIMA-matched renal allografts (2), neonatal heart block (3), type 1 diabetes (4), and cross-generational reproductive fitness with expanded accumulation of NIMA-specific Tregs (5). Here, we considered whether MMcs may confer other physiological benefits beyond these immunological features linked with antigenicity.

A provocative consideration is whether phenotypically WT MMcs can reduce disease severity in autosomal recessive disorders caused by defective or missing proteins. Given a shared susceptibility to infection caused by complement deficiency in humans and mice and enriched MMcs in the liver, where C3 and other complement components are produced, this hypothesis was investigated by evaluating complement levels and infection susceptibility of C3 NIMA mice (C3^–/–^ mice born to C3^+/–^ mothers) compared with genetically identical C3^–/–^ mice born to complement-deficient mothers, along with C3^+/–^ littermate controls (Figure 1A and Supplemental Figure 1; supplemental material available online with this article; https://doi.org/10.1172/JCI187001DS1).

We found increased serum C3 levels in C3 NIMA mice compared with C3^–/–^ mice born to complement-deficient mothers, albeit at levels still considerably reduced compared with levels in C3^+/–^ controls (Figure 1B). C3^–/–^ mice are highly susceptible to *E. coli*, and an intermediate dosage (15,000 CFU) that accentuates this susceptibility was used for intravenous infection to further investigate the functional consequences of complement-producing MMcs (Supplemental Figure 2). These experiments showed that the normally high bacterial burden in tissues of infected C3^–/–^ mice were reduced in C3 NIMA mice (Figure 1C), demonstrating that being born to complement-sufficient mothers can dominantly affect infection susceptibility of otherwise genetically identical complement-deficient mice.

To verify importance of C3^+/–^ MMcs, we evaluated C3 levels and infection susceptibility after MMc depletion using antibody- or pregnancy-induced MMc displacement in male and female C3 NIMA mice, respectively. For antibody MMc depletion, transgenic mice with constitutive cell-surface expression of OVA (5, 6) were intercrossed with C3^–/–^ mice to generate C3 OVA NIMA offspring born to C3^+/–^ OVA^+/–^ mothers (Supplemental Figure 3). Transforming OVA with C3 into NIMAs in this fashion allowed MMc depletion using anti-OVA IgG, and verification of loss of MMc by quantifying OVA^+^ genomic DNA in tissues such as heart, liver, and uterus, which consistently contain the highest MMc levels (5, 6). These experiments showed that C3^+/–^ OVA^+/–^ MMc depletion reduced serum C3 to background levels (Figure 1B) and overturned infection resistance of C3 NIMA mice (Figure 1, C and D).

Despite the ability to persist long term, MMcs are also susceptible to pregnancy-induced displacement and replacement with fetal microchimeric cells (FMcs) (6). To further investigate the necessity of C3^+/–^ MMcs in C3 NIMA females, we compared C3 levels and susceptibility after pregnancy sired by complement-deficient males with ensuing replacement by C3^–/–^ FMcs. C3 NIMA female mice postpartum after pregnancy sired by C3^–/–^ males, with loss of C3^+/–^ MMcs, contained only background serum C3 levels (Figure 1B) and infection susceptibility comparable with C3^–/–^ controls (Figure 1, E and F). Thus, complement-producing MMcs were responsible for the above-background C3 levels and reduced infection susceptibility in complement-deficient offspring.

Beyond complement deficiency, these results suggesting clinical phenotypes associated with missing or defective proteins in autosomal recessive disorders can be altered by functionally WT MMcs open up fundamental new ways for explaining why individuals with the same gene defect in many autosomal recessive disorders, including cystic fibrosis and sickle cell anemia, have widely varied disease severity. In turn, these protective benefits associated with complement-producing MMcs highlight the importance of further investigating how these cells work, including their cellular identity and phenotype heterogeneity, since expanding their accumulation beyond natural microchimeric levels represents an innovative approach for therapeutically reducing the severity of common genetic disorders.

## Supplementary Material

Supplemental data

Supporting data values

## Figures and Tables

**Figure 1:**
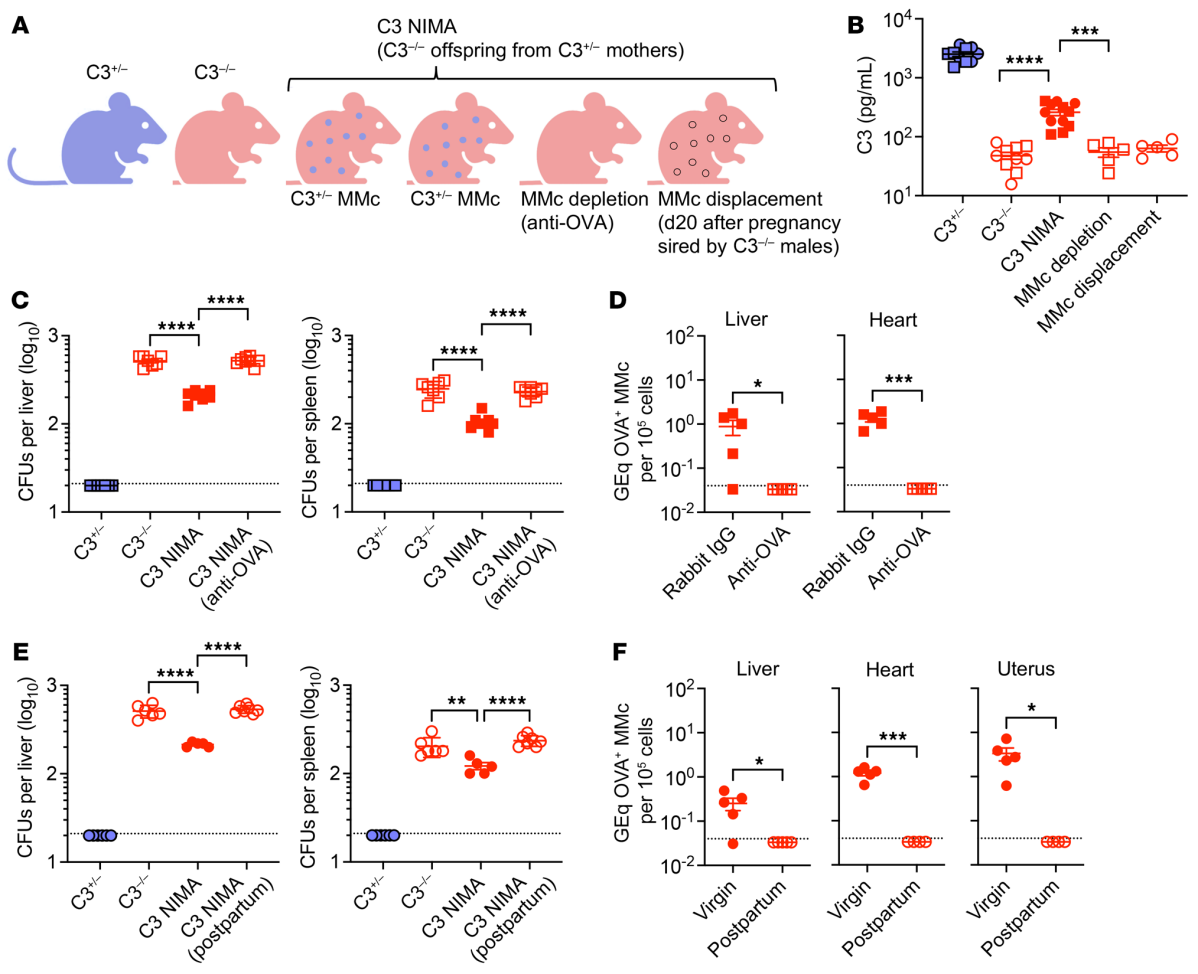
C3^+/–^ MMcs override complement deficiency in C3^–/–^ mice. (**A**) Schematic comparing C3^+/–^, C3^–/–^, and C3 NIMA mice, or C3 NIMA mice after MMc depletion or displacement. d20, day 20. (**B**) Serum C3 levels in the male (square) or female (circle) mice described in **A**. (**C**) *E. coli* CFU after infection for male C3^+/–^, C3^–/–^, C3 NIMA, and C3 NIMA mice depleted of OVA^+^ MMcs using anti-OVA IgG. (**D**) Genome equivalent (GEq) OVA DNA specific to OVA^+^ MMcs in male C3 OVA NIMA mice 14 days after anti-OVA compared with isotype control IgG administration. (**E**) *E. coli* CFU after infection for female C3^+/–^, C3^–/–^, virgin C3 NIMA, and C3 NIMA mice postpartum after pregnancy sired by C3^–/–^ males. (**F**) GEq OVA DNA specific to OVA^+^ MMcs among female C3 OVA NIMA mice 20 days postpartum after pregnancy sired by C3^–/–^ males compared with age-matched virgin control mice. Each point represents data from an individual mouse, combined from at least 2 independent experiments, each with similar results. Data indicate the mean ± SEM. *P < 0.05, **P < 0.01, ***P < 0.005, ****P < 0.001, as determined by 1-way Brown-Forsythe and Welch ANOVA test for comparing data containing more than 2 groups with nonuniform SD (**B**), 1-way ordinary ANOVA for comparing data containing more than 2 groups with similar SD (**C** and **E**), and unpaired Student’s *t* test when comparing 2 groups (**D** and **F**).

## Version 1 11/14/2024

In-Press Preview

## Version 2 01/02/2025

Electronic publication
