# Supplementary material for: Complement-producing maternal microchimeric cells override infection susceptibility in complement-deficient murine offspring
Source: J Clin Invest. 2024 Nov 14;135(1):e187001. doi: 10.1172/JCI187001 (PMC11684811; doi:10.1172/JCI187001)
Supplement: Supplemental data [file jci-135-187001-s070.pdf]

Supplemental Materials

Supplemental Methods

Sex as a biological variable. Our study examined male and female animals, and similar findings are reported for both sexes. Gender-matched controls were used in all experiments, with the data from female and male mice depicted using square and circle symbols, respectively, in each figure panel.

Mice. Inbred H-2b/b C57BL/6 (Jackson stock #000664) and C3-/- on the C57BL/6 background (Jackson stock #029661) were purchased from The Jackson Laboratory, and bred at Cincinnati Children's Hospital. OVA transgenic mice engineered to constitutively express a cell surface recombinant protein containing full-length ovalbumin on the C57BL/6 background have been described (5-8).

Serum C3 quantification. Blood was collected from mice by retroorbital bleeding, allowed to clot spontaneously at room temperature (10 minutes), followed by centrifugation for serum harvest, and storage at -80ºC. Levels of C3 were evaluated using commercial kits (Abcam, cat #ab263884) according to the manufacturer's instructions.

Depletion of OVA:2W1S+ microchimeric cells. For OVA+ MMc depletion, 6-8 week old male C3 NIMA mice were administered 650 µg purified α-OVA (Acris, cat #R1101) or rabbit IgG isotype antibody (Sigma-Aldrich, cat #I8140) by intraperitoneal injection, followed by a second 325 µg treatment using the same antibody 10 days later, and evaluation of serum C3 and OVA+ MMc levels, and *E. coli* infection susceptibility 2-3 days after the second α-OVA or rabbit IgG isotype antibody treatment.

DNA extraction and quantitative PCR. Each tissue were sterilely dissected, and DNA extracted using the QIAamp DNA extraction kit (Qiagen). PCR for enumerating OVA+ DNA was performed in 12 separate wells per tissue each containing 333 ng genomic DNA (GEq ~3.33 x 10^5 cells) in 20 µl total volume together with 10 µl Taqman Gene Expression Master Mix and 1

μl ovalbumin-specific Taqman assay (Applied Biosystems), for a detection limit of ~1 in 4 x 10^6 cells genomic equivalents per tissue as described (5, 6). Amplification was performed using the 7500 Fast Real-Time PCR System (Life Technologies) under the following program: 95°C for 10 minutes, followed by forty cycles of 95°C for 15 seconds and 60°C for 1 minute, and compared with a standard curve for OVA+ as described (5, 6).

Bacteria. For infection, the uropathogenic *Escherichia coli strain* UTI89 was grown to early log phase (OD_{600} 0.1) in brain heart infusion media at 37ºC, washed and diluted to each dosage in sterile saline, and inoculated intravenously via the lateral tail vein as described (9, 10). The inoculum for each experiment was confirmed by spreading a diluted aliquot onto agar plates. Twenty four hours after infection, the number of recoverable bacteria was evaluated by sterilely dissecting the liver and spleen from infected mice, homogenization in saline containing 0.05% Triton X-100, plating serial dilutions of each tissue homogenate onto agar plates, and enumeration after 37ºC incubation for 24 hours.

Statistical analysis. *P* values were determined using one way Brown-Forsythe and Welch ANOVA test for comparing data containing more than two groups with non-uniform standard deviation (Figure 1B), one way ordinary ANOVA for comparing data containing more than two groups with similar standard deviation (Figure 1C, 1E), or unpaired Student's t test when comparing two groups (Figure 1D and 1F). All tests were performed with Prism 10 (GraphPad).

Study approval. All experiments using age- and gender-matched controls were performed under Cincinnati Children's Hospital Research Foundation IACUC approved protocols (Cincinnati, OH, USA).

Data availability. A single Microsoft excel file containing values for all data shown in the primary and supplementary figures is provided in the Supporting Data file.

Acknowledgements: We are grateful to members of the Way laboratory, and Center for Inflammation and Tolerance faculty for helpful discussions, veterinary and administrative staff in Cincinnati Children's Hospital Research Animal Resources for outstanding animal care. This

work was supported in part by NIH-NIAID through grants R01-AI175431 and R01-AI184537, the Burroughs Wellcome Fund Investigator in the Pathogenesis of Infectious Disease Award (grant #1011031) and March of Dimes Ohio Prematurity Research Collaborative. L.H.T. was supported by the NIAID through Vaccinology Training Program NIH-NIAID T32-AI165396. This work illustrating new maternal benefits is dedicated to Mrs. Viola Eldora Westlund Putnam (1929-2024), mother to seven children, and countless number of grandchildren and great grandchildren, including the children of corresponding author S.S.W.

Author contributions: Designing research (all authors), conducting experiments (G.P.; R.E.D.), Analyzing data (G.P.; R.E.D.; S.S.W.), writing manuscript (all authors)

Supplemental References

7. Ehst BD, Ingulli E, and Jenkins MK. Development of a novel transgenic mouse for the study of interactions between CD4 and CD8 T cells during graft rejection. *Am J Transplant.* 2003;3(11):1355-62.
8. Moon JJ, Dash P, Oguin TH, 3rd, McClaren JL, Chu HH, Thomas PG, et al. Quantitative impact of thymic selection on Foxp3+ and Foxp3- subsets of self-peptide/MHC class II-specific CD4+ T cells. *Proc Natl Acad Sci U S A.* 2011;108(35):14602-7.
9. Elahi S, Ertelt JM, Kinder JM, Jiang TT, Zhang X, Xin L, et al. Immunosuppressive CD71+ erythroid cells compromise neonatal host defence against infection. *Nature.* 2013;504(7478):158-62.
10. Prasanphanich NS, Gregory EJ, Erickson JJ, Miller-Handley H, Kinder JM, and Way SS. Preconceptual Priming Overrides Susceptibility to Escherichia coli Systemic Infection during Pregnancy. *mBio.* 2021;12(1).

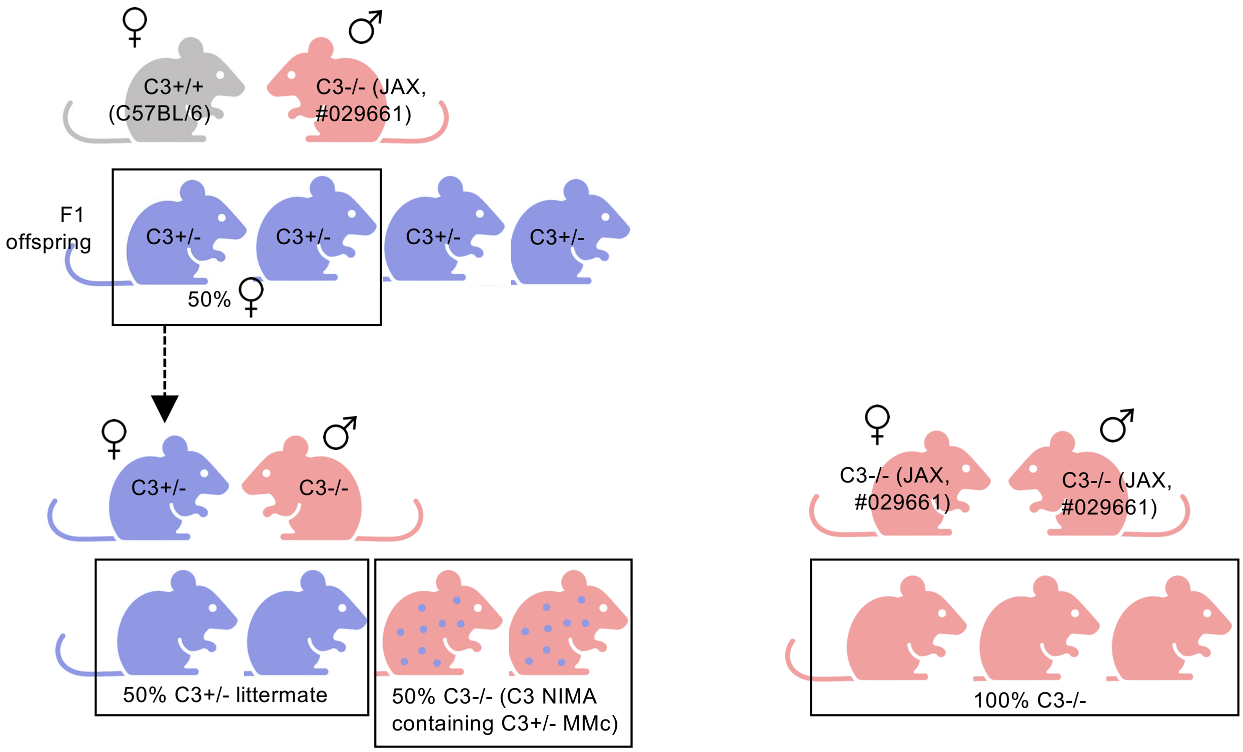

Supplemental Figure 1. Breeding scheme for generating genetically identical C3-/- mice born to C3+/- mothers (C3 NIMA) (left) compared with C3-/- mothers (right), and C3+/- littermate control mice.

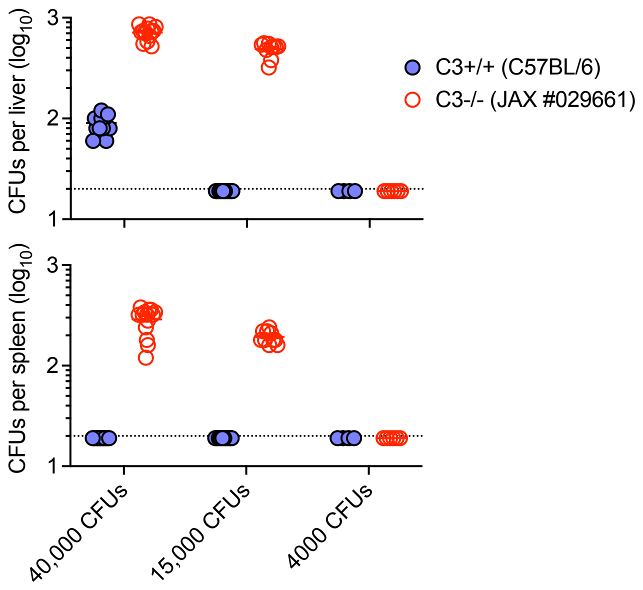

Supplemental Figure 2. Recoverable *E. coli* CFUs in the liver and spleen of C57BL/6 wildtype (C3+/+) compared with isogeneic C3-/- mice 24 hours after intravenous infection with each dosage of uropathogenic *E.* coli strain UTI89. Each point represents the data from an individual mouse, combined from 2 independent experiments each with similar results.

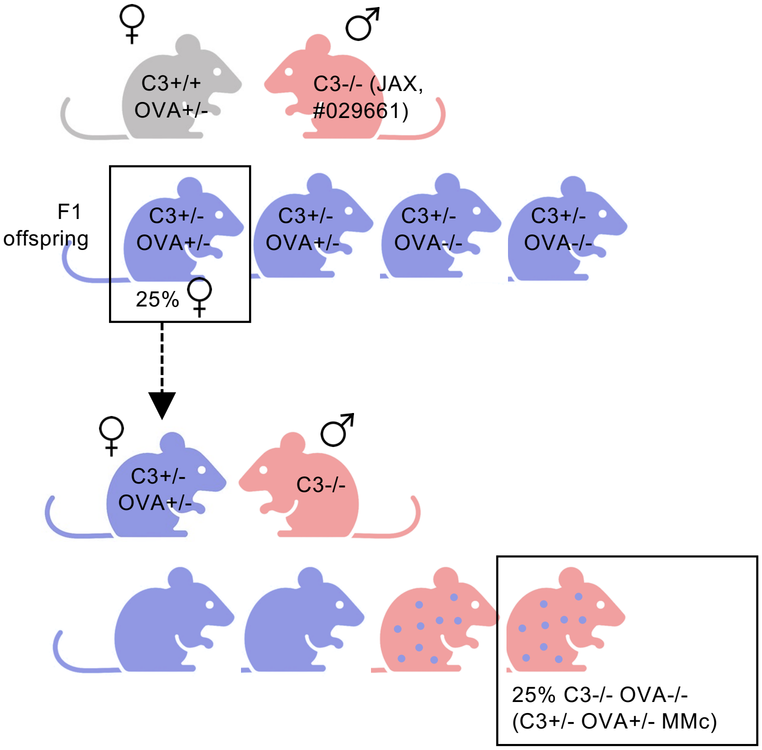

Supplemental Figure 3. Breeding scheme for generating C3-/- OVA-/- mice born to C3+/- OVA+/- mothers (C3 OVA NIMA) containing C3+/- OVA+/- MMc susceptible to depletion using anti-OVA IgG.
